# A Medical-Grade Polycaprolactone and Tricalcium Phosphate Scaffold System With Corticoperiosteal Tissue Transfer for the Reconstruction of Acquired Calvarial Defects in Adults: Protocol for a Single-Arm Feasibility Trial

**DOI:** 10.2196/36111

**Published:** 2022-10-13

**Authors:** Isabel Gonzalez Matheus, Dietmar W Hutmacher, Sarah Olson, Michael Redmond, Allison Sutherland, Michael Wagels

**Affiliations:** 1 Department of Plastic & Reconstructive Surgery Princess Alexandra Hospital Queenland Australia; 2 Herston Biofabrication Institute Herston Australia; 3 The Australian Centre for Complex Integrated Surgical Solutions Translational Research Institute Woolloongabba Australia; 4 School of Medicine University of Queensland Brisbane Australia; 5 Regenerative Medicine Institute of Health and Biomedical Innovation Queensland University of Technology Kelvin Grove Australia; 6 Department of Neurosurgery Princess Alexandra Hospital Woolloongabba Australia; 7 Department of Neurosurgery Royal Brisbane & Women's Hospital Herston Australia

**Keywords:** 3D printing, reconstruction, cranioplasty, free flap, craniofacial, surgery, bone, skull

## Abstract

**Background:**

Large skull defects present a reconstructive challenge. Conventional cranioplasty options include autologous bone grafts, vascularized bone, metals, synthetic ceramics, and polymers. Autologous options are affected by resorption and residual contour deformities. Synthetic materials may be customized via digital planning and 3D printing, but they all carry a risk of implant exposure, failure, and infection, which increases when the defect is large. These complications can be a threat to life. Without reconstruction, patients with cranial defects may experience headaches and stigmatization. The protection of the brain necessitates lifelong helmet use, which is also stigmatizing.

**Objective:**

Our clinical trial will formally study a hybridized technique's capacity to reconstruct large calvarial defects.

**Methods:**

A hybridized technique that draws on the benefits of autologous and synthetic materials has been developed by the research team. This involves wrapping a biodegradable, ultrastructured, 3D-printed scaffold made of medical-grade polycaprolactone and tricalcium phosphate in a vascularized, autotransplanted periosteum to exploit the capacity of vascularized periostea to regenerate bone. In vitro, the scaffold system supports cell attachment, migration, and proliferation with slow but sustained degradation to permit host tissue regeneration and the replacement of the scaffold. The in vivo compatibility of this scaffold system is robust—the base material has been used clinically as a resorbable suture material for decades. The importance of scaffold vascularization, which is inextricably linked to bone regeneration, is underappreciated. A variety of methods have been described to address this, including scaffold prelamination and axial vascularization via arteriovenous loops and autotransplanted flaps. However, none of these directly promote bone regeneration.

**Results:**

We expect to have results before the end of 2023. As of December 2020, we have enrolled 3 participants for the study.

**Conclusions:**

The regenerative matching axial vascularization technique may be an alternative method of reconstruction for large calvarial defects. It involves performing a vascularized free tissue transfer and using a bioresorbable, 3D-printed scaffold to promote and support bone regeneration (termed the *regenerative matching axial vascularization* technique). This technique may be used to reconstruct skull bone defects that were previously thought to be unreconstructable, reduce the risk of implant-related complications, and achieve consistent outcomes in cranioplasty. This must now be tested in prospective clinical trials.

**Trial Registration:**

Australian New Zealand Clinical Trials Registry ACTRN12620001171909; https://tinyurl.com/4rakccb3

**International Registered Report Identifier (IRRID):**

DERR1-10.2196/36111

## Introduction

### Background

Cranioplasty, as a procedure for reconstructing cranial defects, has been around for thousands of years. Despite significant advances in technology and extensive research into bone biology, the reconstruction of the calvarium still poses a significant challenge for surgeons [1,2]. Since the early 1900s, autologous bone grafts have been considered the gold standard, acting as readily available donor materials with marked strength, elasticity, assured biocompatibility, and the ability to provide adequate cosmesis [3]. Despite this, autologous bone grafts are not without complications, including bone flap resorption, and graft failure rates of up to 35% have been reported [4-7]. Other autologous bone grafts (eg, those in the form of banked allografts or those made of bone harvested from other anatomical locations) have also been shown to result in increased donor site morbidity and higher rates of graft failure.

These issues with autologous bone grafts have prompted research into suitable synthetic alternatives; metals, synthetic ceramics, and polymers are all advantageous in different ways but have significant fallbacks that relate to infection, malleability, and in situ inflammatory or exothermic reactions [8,9]. A bioresorbable composite scaffold comprised of polycaprolactone and tricalcium phosphate (PCL-TCP) has already been shown to be effective in various bone engineering platforms [7,10-13]. Further, 3D computed tomography (CT) modeling has revolutionized the customization of these osteoconductive scaffolds, enabling their seamless integration into skull defects, with aesthetic cranial contouring. Osteoinduction is a process whereby osteoprogenitor cells differentiate into osteoblasts and therefore is crucial in osteogenesis. Within PCL-TCP scaffolds, osteoinduction can be enhanced through the addition of biological additives (eg, bone morphogenetic protein-7) or cellular approaches (eg, the use of mesenchymal osteoprogenitor stem cells) that have been shown to augment bone growth [14-18].

The success of these cellular impregnation techniques for promoting acceptable osteogenesis has been shown to be directly linked with the vascularity of the constructs used [13,18]. The utilization of flap-based tissue with dependable axial vascularization (eg, based on a known arteriovenous system) not only enhances angiogenesis but also provides a specific tissue type that matches the intended regeneration along with corresponding progenitor cells [19]. Axial vascularization via a corticoperiosteal flap to stimulate bone regeneration is an example of regenerative matching axial vascularization (RMAV) [20]. The experimental application of this theory with corticoperiosteal flaps has been implemented in cases of critical long bone defects, with the results indicating enhanced bone regeneration and scaffold integration [21-23].

Throughout the literature, there is a clear consensus that an ideal cranioplasty graft should have the following: (1) long-term mechanical protection for the brain and meninges until the host bone regenerates, (2) acceptable cosmetic contouring, and (3) graft materials that promote bone growth through osteoconductive and osteoinductive mechanisms that are governed by degradation kinetics and biological interactions. Moreover, if the prosthesis remains, it should not cause undue complications or patient inconveniences.

The composite PCL-TCP scaffold acts as a synthetic analogue of two major bone constituents—hydroxyapatite and collagen. As cranioplasty materials, PCL-TCP fulfill the above criteria by retaining strength and promoting osteoconduction over extended remodeling periods via consistent degradation mechanics and with minimal adverse effects. As a consequence of advancements in tissue engineering, the demand for customizable implants is understandably intensifying. This case highlights the potential of harnessing emerging biomedical technologies and 3D tailoring to meet individual and specific clinical needs.

In a case report published by Yogishwarappa et al [24] in 2016, the authors utilized a 3D-printed PCL-TCP scaffold with iliac crest stem cells to reconstruct a craniotomy defect. The authors described good bone growth at 18 months but did not quantify this result via CT imaging, which has traditionally been the case in preceding animal studies. This case went further by incorporating axial vascularization techniques with strategies for the targeted regenerative matching of tissue types and progenitor cells.

Osteoinduction and, as a consequence, osteogenesis are intricately linked to angiogenesis in normal fracture healing [25]. Corticoperiosteal tissue from the medial femoral condyle can provide a vascularized source of tissue rich in osteogenic progenitor cells. Studies have shown that specific osteoprogenitor cells, such as those in periosteal tissue, are stimulated by growth factors to differentiate and form bone [26]. As a result, scaffolds are subject to the uniform delivery of host-derived growth factors, mesenchymal stem cells, and an optimal extra-cellular environment, of which all are essential for bone regeneration.

Investigations into periosteal flaps as sources of axial vascularization are well documented in animal models. Nau et al [22] compared periosteal flaps with a tricalcium phosphate scaffold that did or did not have bone marrow–derived mononuclear cells to a periosteal flap alone. These were used in rat femurs with critical size defects. The periosteal flap with tricalcium phosphate and marrow cells had a significant increase in new bone formation, vascularization and strength radiographic, histological, and biomechanical outcomes at 4 weeks. More recently, Sparks et al [20] combined a pedicled corticoperiosteal flap, which was based on the anterior tibial vessels, with a PCL-TCP scaffold to bridge 3- and 6-cm tibial segmentectomies. The radiological and histological outcomes demonstrated new bone regeneration and excellent scaffold integration, with the complete bridging of both defects at 12 months.

The research by our group at the Centre for Regenerative Medicine (Queensland University of Technology, Brisbane, Australia) has focused on the reconstruction of critical-sized bone defects for more than 1 decade [13,17,27]. In particular, our work has focused on the bench-to-bedside translation of tissue-engineered bone replacements that are biodegradable, ultrastructured composite scaffolds made of medical-grade PCL-TCP (mPCL-TCP) [28]. In vitro, this scaffold system supports cell attachment, migration, and proliferation with slow but sustained degradation to permit host tissue regeneration and the replacement of the scaffold [29]. The in vivo compatibility of this scaffold system is robust [30,31], and this system may be used alongside the use of growth factors, such as recombinant human bone morphogenic protein-7 (rhBMP-7), that are known to promote the regeneration of bone. Importantly, the mPCL-TCP scaffold has been extensively evaluated in preclinical studies with a validated model of a large animal with a critical-sized bone defect that was developed by our group [13]. Our research using this model has shown that this scaffold system, when used alongside rhBMP-7 for a 3-cm, critical-sized long bone defect, is highly regenerative with respect to bones [13].

Although osteoinductive scaffolds have been trialed clinically by a number of authors [32-35], limitations in preclinical work that prevent the mainstream application of this approach remain. Scaffold vascularization, which is inextricably linked to bone regeneration, remains key, and its importance is generally underappreciated [18]. A variety of methods have been investigated to address this issue, including the prevascularization of scaffold prior to transplantation, vessel-based approaches for axial vascularization (arteriovenous loops and associated variations), and flap-based approaches for axial vascularization (muscle flaps, periosteal flaps, etc). Clearly, the ideal method for vascularizing a scaffold would also promote bone regeneration.

The regenerative capacity of vascularized corticoperiosteal tissue is well recognized [36,37]. It has a robust blood supply, and potential donor sites for distant transfer are plentiful [19]. For uniform scaffold vascularization, an intrinsic approach is considered key [38]. However, to date, a combined intrinsic and flap-based approach to scaffold vascularization that also exploits the innate autologous regenerative capacity of corticoperiosteal tissue for bone regeneration has not been explored. We define the coalescence of these concepts as RMAV, whereby a corticoperiosteal flap is used to vascularize scaffold intrinsically and also produce bone.

Between 2016 and 2018, we undertook a preclinical study that evaluated the concept of RMAV by using a corticoperiosteal flap from a sheep’s hind limb and a 3D-printed mPCL-TCP scaffold to reconstruct 6- and 3-cm intercalary defects of the tibia [20]. This technique was compared against existing control data sets for this animal model. Control groups were reconstructed with autologous bone grafts alone, were reconstructed with mPCL-TCP scaffolds alone, or were left unreconstructed [20]. The regenerative matching approach resulted in the enhanced volume of regenerate bone, as shown on plain x-ray and micro-CT images [20], and equivalent biomechanical torsional stiffness when compared to the approach using bone grafts alone. In December 2019, we performed a first-in-human case study of applied RMAV, wherein a 3D-printed mPCL-TCP scaffold with rhBMP-7 and vascularized corticoperiosteal flaps was used to successfully perform a reconstruction of a large calvarial defect that had failed to heal after conventional cranioplasty [39]. This work builds on our experiences with bone defect reconstructive research, and it will involve the regenerative matching technique.

Given these successes in preclinical research and our first-in-human case, we feel that the logical next step for the emerging RMAV technique should be a robust feasibility clinical trial examining the behavior of the mPCL-TCP implant and postimplantation bone healing. Once appropriate outcome measures are clearly defined, it may then be appropriate to proceed to a randomized clinical trial in which RMAV is compared to other clinical techniques for a given bone defect.

### Objectives

#### Hypothesis

The use of an mPCL-TCP scaffold, in conjunction with a corticoperiosteal tissue transfer, is a safe and effective approach to reconstructing critical-sized bone defects in the calvarium.

#### Primary and Secondary Objectives

The primary objectives of our trial are to (1) determine the feasibility and efficacy of using an mPCL-TCP scaffold, in conjunction with a vascularized corticoperiosteal tissue transfer, for the reconstruction of calvarial bone and (2) determine the clinically relevant functional outcomes that follow the reconstruction of an acquired calvarial defect by using an mPCL-TCP scaffold in conjunction with a vascularized corticoperiosteal tissue transfer.

The secondary objective is to collect data to allow for the later evaluation of the cost-effectiveness of this approach and a view of its applications across a broader range of clinical situations.

## Methods

### Study Design

Our study will be a single-arm feasibility trial that evaluates the outcomes of using an mPCL-TCP scaffold, in conjunction with a vascularized corticoperiosteal tissue, in participants with acquired defects of the calvarial bones of the skull resulting from trauma, malignancy, or infection. The study will capture data that will be used to inform the design of future clinical trials in this area.

The mPCL-TCP scaffold that will be used in the study will be manufactured by the trial sponsor—Osteopore International Pte Ltd (Singapore). Osteopore products are made of a US Food and Drug Administration–approved polymer (K051093) called *polycaprolactone*, which is bioabsorbable, is malleable, is slow to degrade, and possesses mechanical strength that is similar to that of trabecular bone. The product will be manufactured by using 3D printing technology that is precise and allows for the customization of shape and geometry. The unique, 3D-printed, biomimetic microarchitecture of the 3D scaffolding, which is contained within Osteopore products, allows for the infiltration of cells and blood vessels, of which both play a key role in wound healing and tissue repair.

### Study Setting

The study will be an open-label, single-arm feasibility trial that will be jointly coordinated by the investigators at the Princess Alexandra Hospital (PAH) in Woolloongabba (Queensland, Australia), the Herston Biofabrication Institute, the Australian Centre for Complex Integrated Surgical Solutions at the Translational Research Institute (Queensland, Australia), and the Centre for Regenerative Medicine at the Institute of Health and Biomedical Innovation in Kelvin Grove (Queensland, Australia). With the aim of calvarial reconstruction, the study population will include any patient with a calvarial defect that is amenable to reconstructive approaches that involve using the Osteopore implant, following discussions by the multidisciplinary team (MDT) of investigators.

### Eligibility Criteria

#### Inclusion Criteria

Our inclusion criteria are as follows:

- Acquired defect of the calvarium that is suitable for reconstruction by using a PCL-TCP scaffold system and performing a corticoperiosteal tissue transfer
- Patients aged >18 years and <55 years
- Patients aged >55 years may still be eligible for the trial after an assessment by and at the discretion of the investigators, and documentation to this effect will be provided by the clinic for the trial documents pertaining to such patients
- Patients who are willing and able to comply with the study requirements
- Patients who are eligible for undergoing magnetic resonance imaging (MRI; ie, no implanted metal or metal devices and no history of severe claustrophobia)
- Patients or their guardians are capable of providing valid informed consent

#### Exclusion Criteria

Our exclusion criteria are as follows:

- Active infection at the time of study inclusion (in chronic infection cases, active infections may manifest as the result of a failed trial of being taken off antibiotics)
- Patients or their guardians are unwilling or unable to provide fully informed consent
- Patients with a known history of immunodeficiency, including a history of HIV, concomitant systemic corticosteroid therapy, chemotherapy, synchronous hematological malignancy, or other causes of a secondary or primary immunodeficiency
- Patients with a known severe concurrent or intercurrent illness (eg, a cardiovascular, respiratory, or immunological illness; psychiatric disorders; alcohol or chemical dependence; or possible allergies [including allergy to polycaprolactone]) that would, in the opinion of the primary investigator, compromise their safety or compliance or interfere with the interpretation of study results
- Women who are currently pregnant, are breastfeeding, or are planning to become pregnant within 2 years after the reconstruction surgery
- Women of childbearing potential who are not using an appropriate contraceptive method
- Patients who are ineligible for undergoing MRI
- Patients with a life expectancy of <36 months.
- Patients who are unable or unwilling to comply with the study requirements

### Sample Size

This feasibility trial will recruit a total of 10 patients.

### Recruitment

As a part of their clinical management, patients with acquired calvarial defects from the neurosurgery clinic or the skull base/head and neck clinic at the PAH will be reviewed as candidates for cranial reconstruction via the Osteopore implant by the MDT. Referrals to this process will be accepted from throughout Queensland via standard pathways, and national out-of-catchment referrals will be considered for special circumstances in which the PAH has unique expertise. The specific conditions assessed may include acute and subacute pathologies (major acute fractures with bone loss, bone malignancy, etc) or chronic pathologies (osteomyelitis, osteoradionecrosis, etc).

The relevant specialties that will be involved in the MDT review and decision-making process include infectious diseases, plastic and reconstructive surgery, neurosurgery, infectious diseases, and engineering. The MDT will determine the best treatment option for each patient. Should cranial reconstruction by using an Osteopore implant be recommended by the MDT, the patient’s treating specialist will discuss the case with the coordinating principal investigator, and the extent to which the potential participant satisfies the trial’s inclusion and exclusion criteria will be determined collaboratively. Written informed consent will be obtained following the MDT’s decision and prior to the conduct of the cranioplasty. Informed consent must be completed prior to any study-related activities.

Patients will be followed up for at least 24 months from the time of initial reconstruction. Given that the PAH collectively sees around 5 patients per year with significant bone defects, the recruitment phase will take place over 2 to 3 years to recruit a total of 10 patients for the feasibility analysis.

In addition to the prospective recruitment pathway detailed above, patients who have undergone cranioplasty via the Osteopore implant may also be recruited into the study at the discretion of the sponsor and coordinating principal investigator.

### Intervention

Prior to deciding on a treatment pathway, medical imaging will be conducted as a part of each patient’s standard clinical management, which may include CT and/or MRI scans of the head and neck, as well as white cell–labeled, whole-body bone scans. The imaging results will inform the clinical assessment performed by the neurosurgeon or the skull base/head and neck surgeon involved in the care of the patients and inform the MDT review. Clinical notes, including medical histories, will also be generated as a part of the clinical care process of patients during their clinical management. Imaging data and clinical notes that relate to the defects among patients and are collected prior to informed consent will be made available to the study participants as a part of the informed consent process.

**Figure 1:**
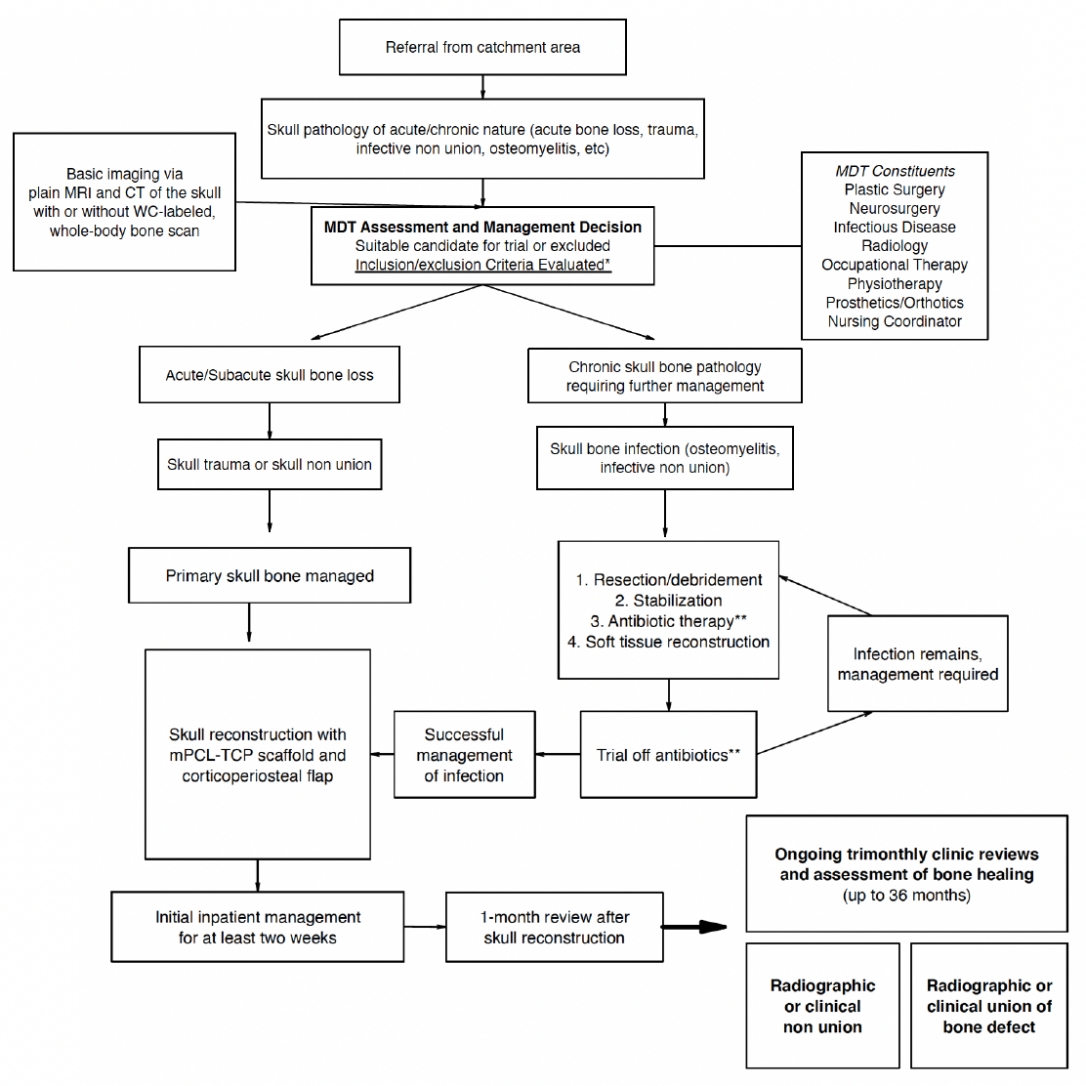
Flowchart of patient journey. CT: computed tomography; MDT: multidisciplinary team; mPCL-TCP: medical-grade polycaprolactone and tricalcium phosphate; MRI: magnetic resonance imaging; WC: white cell. *see "Eligibility Criteria" section; **antibiotic therapy, as indicated by infectious disease physician consultation.

A description of the patient journey is summarized in Figure 1. The clinical care reconstruction will be performed at time 0. A standard clinical protocol for wound care and rehabilitation will follow. Patients may undergo a review for routine clinical assessments every 3 months following the cranioplasty until they are clinically stable, with more or less frequent follow-up visits arranged according to their clinical needs.

The trial-related interventions will focus on capturing defined functional outcomes and, consequently, will require the collection of data at specific time points. CT and/or MRI scans of the head and neck will be used to identify complications and establish clinical and radiological evidence of union at 1, 12, and 24 months after surgery. As performing 2 CT scans in the first year during the postoperative follow-up period is standard clinical practice, the amount of ionizing radiation to which the participants are exposed will not exceed the amount of radiation that patients typically receive during standard clinical care. Additional CT and MRI scans may be performed during the follow-up period in accordance with patients’ clinical needs.

The other research-related functional assessments will include the administration of the Quality of Life After Brain Injury (QOLIBRI) and 36-item Short Form Quality of Life (SF-36) questionnaires at prereconstruction and at 1, 12, and 24 months after surgery.

### End Points

The key primary end points for evaluation in our single-arm study are the time to the formation of stable, regenerated bone that is sufficient for protecting the brain (ie, radiographic or clinical evidence of bony union) and patient-reported outcomes. These primary end points serve as surrogate markers for satisfactory bone healing but, more importantly, are clinically useful for and relevant to the study goal and overarching purpose of the intervention. Functional outcomes are more difficult to measure reliably and are therefore secondary outcomes, which will be measured with the QOLIBRI and the SF-36 questionnaires [40]. The schedule of assessments is presented in Table 1.

**Table 1:** Schedule of assessments.

			Multidisciplinary team review		Enrollment		Reconstruction		Follow-up period		
									1-month review		3-, 6-, 9-, 12-, 15-, 18-, 21-, and 24-month review
Time point^a^			−t_2_		−t_1_		0		T_1_		T_2-9_
**Enrollment**											
	Informed consent			✓^b^							
	Eligibility screen			✓							
	Demographics			✓							
	Medical history and medical review	✓		✓				✓		✓	
**Interventions**											
	Infection management			✓							
	Reconstruction					✓					
**Assessments**											
	Computed tomography of head	✓						✓		✓^c^	
	Magnetic resonance imaging	✓						✓		✓	
	White cell–labeled, whole-body bone scan	✓						✓		✓	
	Quality of Life After Brain Injury questionnaire			✓				✓		✓^c^	
	36-item Short Form Quality of Life questionnaire			✓				✓		✓^c^	

^a^“t” represents a time point prior to the intervention, and “T” represents a time point after the intervention.

^b^Denotes a clinical activity from which data may be utilized for the trial analysis.

^c^Trial-related assessment at 12 and 24 months only.

### Statistical Methods

The statistical analysis of the data will be undertaken by the Queensland Facility of Advanced Bioinformatics (QFAB). QFAB personnel will use REDCap (Research Electronic Data Capture) software (Vanderbilt University) to design and validate an electronic data capture system for use in the trial. Deidentified data will be entered into the REDCap-based electronic data capture system by a member of the research team after the coordinating principal investigator or delegate has certified each case report form (CRF). The database will contain validation ranges for each variable to minimize the chance of data entry errors. An audit trail will be created to maintain a record of the initial entries and changes made, the reasons for changes, the times and dates of entries, and the usernames of the people who made the changes. Data queries will be raised, and missing data or suspected errors will be resolved prior to locking the database and performing the analysis.

### Analysis

All patients who are registered for the trial will be accounted for in the (intention-to-treat) analysis. For retrospectively recruited patients, all data points may not be available for all assessments, or data collection may have occurred outside of the trial-specific window (eg, CT data are available for the 9-month follow-up but are not available for the 12-month follow-up). In such cases, these subjects will not be evaluated for a particular analysis unless the number of data points required for the analysis is enough, or a trend can be extrapolated from the data that were collected outside of the trial-specified window.

A quantitative analysis that relates to the evaluation of the cost-effectiveness of this approach will not be undertaken as a part of this study, as the data set will most likely be too small to achieve statistical significance. Instead, the outcome measures that have an impact on the financial burden for treating patients will be combined with data from other similar trials that are conducted in the future, and the analysis of health economics data will be undertaken at that point.

Statistical analyses of descriptive statistics for the demographic, primary, and secondary outcomes at each time point will be conducted. A comparison analysis of the questionnaire results at each time point and a univariate Cox regression analysis of the time to weight-bearing for the affected limbs will be performed due to the small sample size. Statistical significance will be defined as *P*<.05. Data will be analyzed by using SPSS for Windows version 22 (IBM Corporation).

### Measures for Avoiding and Limiting Bias

The study represents a high-quality clinical study in which bias will be kept to an absolute minimum. The study will be heavily controlled with the well-defined inclusion and exclusion criteria that will ensure that the number of patients with confounding features will be reduced, thereby effectively minimizing sources of selection bias and increasing the homogeneity of the cohort under investigation.

The follow-up period is 24 months. Generally, we would expect to see sufficient bone regeneration for most extensive defects by this time. Ongoing care will be provided in accordance with the recommendations of the treating surgeons and other members with relevant specialties, as appropriate.

### Ethics Approval

The trial will be conducted in accordance with the principles of the Declaration of Helsinki, Notes for Guidance on Good Clinical Practice (CPMP/ICH/135/95), as adopted by the Australian Therapeutic Goods Administration in 2000, and in accordance with the National Health and Medical Research Council’s National Statement on Ethical Conduct in Research Involving Humans [41]. A copy of the signed and dated letter of approval will be provided to the clinical trial site and the sponsor prior to study commencement. Any written information and advertisements that are to be used for subject recruitment will be approved by the Human Research Ethics Committee prior to their use.

It is the responsibility of the investigator to obtain written informed consent from each individual who participates in the study after an adequate explanation (in lay terms) of the aims; methods; objectives; and potential discomforts, risks, and benefits of the study is given. The investigator must also explain to the subjects that they are completely free to refuse to enter the study or withdraw, without prejudice, from the study at any time. Before enrollment into the study, each prospective candidate will be given a full explanation of the study. Individuals who are eligible and wish to participate will be provided the trial’s participant information and consent form (PICF) prior to participation. The PICF details all of the relevant aspects of the trial procedures. Potential participants will be given time to read through the information and ask any questions. The formal consent process will be undertaken by the principal investigator or the associate investigator and will be documented by the trial coordinator. The PICF will be submitted for approval to the Human Research Ethics Committee of the PAH. Participants will be provided with a copy of their signed PICF.

## Results

As of December 2020, we have enrolled 3 participants for the study. We expect to have results before the end of 2023.

## Discussion

### Study Overview

We aim to evaluate the potential of tissue engineering with a bioresorbable, 3D-printed scaffold and autologous vascularized tissue transfer in the reconstruction of acquired calvarial defects. It is essential to undertake a prospective feasibility trial that formally explores this technique to establish its safety and efficacy. This trial will offer critical baseline data against which current and future implants can be compared. Tissue regeneration could be enhanced via scaffold refinements, including additives such as growth factors, osteoprogenitor cells, and vascular networks [35-39]. However, RMAV may always be necessary to optimize tissue regeneration.

The project, as a whole, has a number of benefits for both individuals and society at large. It is expected that the results of our open-label feasibility trial will provide additional evidence on the effectiveness of the RMAV treatment approach for patients with calvarial defects for which contemporary surgical management may have a greater risk of failure or complications [42,43].

### Conclusion

The RMAV technique may be an alternative method of reconstruction for large calvarial defects. It involves performing a vascularized free tissue transfer and using a bioresorbable, 3D-printed scaffold to promote and support bone regeneration (termed the *regenerative matching axial vascularization* technique). This technique may be used to reconstruct skull bone defects that were previously thought to be unreconstructable, reduce the risk of implant-related complications, and achieve consistent outcomes in cranioplasty. This must now be tested in prospective clinical trials.

## Abbreviations

CT: computed tomography
CRF: case report form
MDT: multidisciplinary team
MRI: magnetic resonance imaging
mPCL-TCP: medical-grade polycaprolactone and tricalcium phosphate
PAH: Princess Alexandra Hospital
PCL-TCP: polycaprolactone and tricalcium phosphate
PICF: participant information and consent form
QFAB: Queensland Facility of Advanced Bioinformatics
QOLIBRI: Quality of Life After Brain Injury
REDCap: Research Electronic Data Capture
rhBMP-7: recombinant human bone morphogenic protein-7
RMAV: regenerative matching axial vascularization
SF-36: 36-item Short Form Quality of Life
